# Effectiveness of acute in-hospital physiotherapy with knee-extension strength training in reducing strength deficits in patients with a hip fracture: A randomised controlled trial

**DOI:** 10.1371/journal.pone.0179867

**Published:** 2017-06-29

**Authors:** Lise Kronborg, Thomas Bandholm, Henrik Palm, Henrik Kehlet, Morten Tange Kristensen

**Affiliations:** 1 Physical Medicine and Rehabilitation Research – Copenhagen (PMR-C), Department of Physio- & Occupational Therapy, Copenhagen University Hospital, Hvidovre, Denmark; 2 Clinical Research Centre, Copenhagen University Hospital, Hvidovre, Denmark; 3 Department of Orthopaedic Surgery, Copenhagen University Hospital, Hvidovre, Denmark; 4 Section for Surgical Pathophysiology, Rigshospitalet, Copenhagen University, Copenhagen, Denmark; University of Sydney, AUSTRALIA

## Abstract

**Question:**

Is acute in-hospital physiotherapy with additional progressive knee-extension strength training (ST) of the fractured limb more effective in reducing knee-extension strength deficit at follow-up compared to physiotherapy without strength training in patients with a hip fracture?

**Design:**

Assessor blinded, randomised controlled trial with intention-to-treat analysis.

**Participants:**

90 patients with a hip fracture admitted to an acute orthopaedic Hip Fracture Unit at a university hospital between October 2013 and May 2015.

**Intervention:**

Daily physiotherapy with or without progressive knee-extension strength training (10RM), 3 x 10 repetitions, of the fractured limb using ankle weight cuffs conducted by ward physical therapists during hospital stay.

**Outcome measures:**

Primary outcome was the change in maximal isometric knee-extension strength in the fractured limb in percentage of the non-fractured limb from inclusion to postoperative day 10 or discharge (follow-up). Secondary outcome was Timed Up and Go test measured early after surgery and at follow-up.

**Results:**

In the intention-to-treat analysis of between-group differences, the primary outcome improved 8.1% (95% CI -2.3; 18.4) by additional strength training from baseline to follow-up. In the per-protocol analysis of non-missing data, significant between-group improvements by 10.5% (95% CI 0.3; 20.7) were found in favour of additional ST. No significant between-group differences were found in any secondary outcome.

**Conclusion:**

Physiotherapy with addition of 5 sessions of ST yielded no additional improvements compared to physiotherapy without strength training in reducing the knee-extension strength deficit at follow-up in patients with a hip fracture. It is debatable whether larger improvements than the observed 8–10% can be expected given that only five exercise sessions, on average, were completed. In fragile patients with a hip fracture in the acute phase, where the ability to participate in functional exercise is compromised, we still consider early strength training a possibility to improve outcomes of clinical importance, given the results of the per-protocol analysis. The present data provides an important basis and call for future investigations including longer term interventions.

**Trial registration:**

Clinicaltrials.gov NCT00848913

## Introduction

Patients with a hip fracture (HF) experience knee-extension strength deficit in the fractured limb of more than 50% compared to the non-fractured limb [1–3] and impaired physical function immediately after HF surgery [4]. This impairment of strength and mobility contributes to further physical deterioration leading to the established long term loss of physical function [5–8], compromised quality of life [9,10], high risk of new falls and fractures [11–13], change of residence [7] and high mortality seen after HF [14,15]. The general aim of postoperative physiotherapy (PT) treatment is for the patient to regain physical function and strength towards the prefracture level of activity; however, the optimal treatment is still not established [16,17]. Despite this, a recent systematic review with meta-analysis and meta-regression provides some evidence for a positive effect on mobility of structured exercise interventions including progressive strength training (ST) after HF [18]. However, these findings are primarily based on studies evaluating prolonged outpatient interventions [18]. The efficacy of very early interventions including standard care rehabilitation are much less studied in patients with HF, although promising results have been found after hip replacement surgery [19] and recently regarding the feasibility of ST initiated within the first days after HF surgery [20]. Consequently, it remains unclear if systematic ST in the acute ward as a supplement to functional exercises, such as walking, rising from a chair and stairclimbing, can reduce the strength deficit in the fractured limb. The hypotheses of this study were that it would be beneficial to receive additional ST in means of reducing the early knee-extension strength deficit and that this reduction may lead to a less impaired physical function early after HF surgery.

Therefore, the purposes of this study in patients with HF were to examine whether: 1) acute in-hospital PT with addition of progressive knee-extension ST of the fractured limb is more effective in reducing the knee-extension strength deficit at follow-up compared to PT without additional ST and, 2) if patients following the ST intervention present larger improvements in physical function compared to PT only.

## Materials and methods

### Design

The study was a randomised, assessor-blinded effectiveness study with parallel assignment to PT with or without ST after HF surgery following our initial feasibility study [20]. All participants received written and oral information about the study, gave written informed consent before enrolment and completed baseline measurements, all performed by the blinded data-assessor. Data was collected at baseline 1–3 days after surgery and at discharge or postoperative day 10 (follow-up). Participants were randomly allocated to two different postoperative in-hospital rehabilitation interventions by a neutral person (blinded to outcomes and patient characteristics) via a computer-generated list with notes placed in sealed envelopes and marked with participant numbers only. The randomisation was stratified for fracture type (trochanteric/femoral neck) to obtain a balanced distribution of fracture types between groups since fracture type has been found to influence physical performance after HF [1,21]. Allocation was concealed to the data-assessor who was also blinded to all baseline data (archived in a locked cabinet) until end of the study. Due to the nature of the intervention, the therapists conducting the ST and the participants were not blinded to the group assignment. After baseline measurements, the physiotherapist responsible for training the participant was handed the sealed allocation envelope by the data-assessor. The therapists and participants were repeatedly instructed not to inform the data-assessor of the assigned treatment and that therapists address questions regarding intervention treatment to a designated neutral consultant. The reporting of the study follows the Consolidated Standards of Reporting Trials (CONSORT) Statement, using the CONSORT Checklist [22] (S1 Table) and the Template for Intervention Description and Replication (TIDieR) checklist [23]. The study protocol, available as supporting information (S1 Text), was approved by the Capital Region’s Research Ethics Committee (H-A-2007-0127 + 37036), registered at clinicaltrials.gov (NCT00843913) https://clinicaltrials.gov/ct2/show/NCT00848913?term=00848913&rank=1 and conducted according to the principles in the Declaration of Helsinki. The individual pictured in this manuscript has given written informed consent (as outlined in PLOS consent form) to publish these case details.

### Changes to method after trial commencement

The stratification on fracture type was violated in the last 10 included patients due to slow inclusion of participants with a femoral neck fracture, allowing more participants with trochanteric fractures (n = 52) to enter the study compared to femoral neck fractures (n = 38).

### Participants, therapists, centres

Participants were recruited from patients admitted to an acute orthopaedic HF bed ward at a University hospital from October 2013 to May 2015. All patients followed a multimodal fast-track programme [24] with the preoperative epidural kept until the 4th postoperative day [25]. The inclusion criteria were home-dwelling patients with a primary HF surgery, aged 65 years or older, able to speak and understand the Danish language, who had given informed consent and with an independent prefracture indoor walking ability equal to a New Mobility Score ≥2 [26]. The exclusion criteria were multiple fractures, weight bearing restrictions, patients unwilling to participate in appropriate rehabilitation or unable to cooperate in tests, terminal illness, and treatment with total hip arthroplasty or parallel pins. The latter were omitted due to the expected short length of hospital stay.

The ST intervention was conducted by 8 regular ward physiotherapists during weekdays and by 10 interchanging physiotherapists during weekends. All physiotherapists involved were skilled in the treatment of orthopaedic patients and specifically trained by the primary investigator in conducting the ST intervention. Instruction of the intervention procedure and recording of data were continuously given during the study with three primary instructions to all involved physiotherapists and a number of individual re-instructions on demand. The outcome measures were collected by the principal investigator, a physiotherapist of more than 10 years of clinical experience, from the Department of Physical Therapy at a University hospital.

### Interventions

The PT treatment in the intervention group (ST group) consisted of the routine PT treatment and additional daily individual progressive knee-extension ST with 3 sets of 10 repetitions performed with an intensity of 10 repetition maximum (10RM), defined as ±2RM of the fractured limb using ankle weight cuffs, as described in detail in our feasibility study [20]. The ST intervention consisted of 5 knee-extensions for each limb separately as a warm up-exercise and with no loads applied. A weight-cuff supposedly matching the patient’s initial level of 10RM was attached around the patient’s ankle of the fractured limb. The ST loads were adjusted on a set-to-set basis and 1-minute pauses separated the sets. The exercise was stopped at a maximum of 15 or less than 8 repetitions in a set and loads increased or decreased respectively for the following set [20]. The routine PT treatment consisted of basic mobility and exercise therapy primarily aimed at lower extremities following a guideline with 12 specific exercises (S2 Text), progressed individually and conducted daily on weekdays with 1–2 contacts per day (weekends included postoperative day 1–3 only). Repetitions and intensity in the routine PT were not standardised. In conjunction with the specific programme, the patients performed exercises consisting of basic mobility activities, balance and stair climbing aimed at regaining physical function corresponding with levels of prefracture habitual activity. Walking aids were changed according to the patient’s level of independent mobility. The PT treatment was performed both as bedside exercise and in the gym located in the HF ward. Participants in the physiotherapy group (PT group) received the routine PT treatment as described above but without any form of ST.

### Outcome measures

Baseline data for age, sex, bodyweight, prefracture physical function (the modified New Mobility Score) [26], mental status (Mini Mental State Examination) [27], the American Society of Anaesthesiologists (ASA) score [28], fall history and type of fracture were collected from medical records and clinical evaluation of the patients.

Primary outcome: Change in maximal isometric knee-extension strength (Nm/Kg) in the fractured limb in percentage of non-fractured limb (Maximal Voluntary Torque (MVT) per kilo body mass) from baseline to follow-up, measured by the reliable method [29] using a belt-fixed handheld dynamometer (Power Track II Commander; JTech Medical, Utah, USA) as previously described in the study by Kronborg et al [20]. Maximal isometric knee-extension strength was assessed with the patient seated on the bedside, hips and knee joint angle in 90° flexion and hands placed on the mattress for support. The lever arm length was measured by tape measure between the lateral epicondyle of the femur and the center of the dynamometer transducer pad placed 4 cm above the lateral malleolous of the tibeal bone. Four trials were completed for each limb of which the greatest value in Newton (N) was used for the analysis. The isometric knee-extension strength was expressed in Nm/kg which was derived from the units of force measured in Newton (N) multiplied by the corresponding lever arm measured in meters (m), divided by the weight (kg) of the patient.

Secondary outcomes: Timed up and go test (TUG) measured in seconds as early as possible after surgery based on the participant having achieved independent walking skills with a rollator and at follow-up using a rollator as a standardised walking aid [30] and standardised instructions [31].

Supplementary outcomes: Gait speed was measured in m/s by the 10-metre fast speed walking test (10 MWT) at follow-up. The Short Falls Efficacy Scale-International (ShortFES-I) [32] was used to measure the patient's fear of falling at follow-up (Score 7–28, high scores indicating a high fear of falling). Basic mobility capacity was measured daily by the Cumulated Ambulation Score (CAS, score 0–6) [33] together with the postoperative day of independent mobility (CAS = 6) [1]. HF-related pain at rest and during ST and outcome assessment was evaluated by Verbal Ranking Scale (VRS, score 0–4) [34], using the highest pain level reported from each session in analysis. The 24-hour time spent upright (minutes per 24hours) from inclusion to follow-up was measured by an activity monitor (ActivPAL3, PAL Technologies Ltd, Glasgow, Scotland) [35]. Time frame of data used in the analysis was from the first complete 24hour data sample to day 6 after that or the last day of data sampling if discharged earlier. The content of the routine PT treatment was recorded daily with number of repetitions in the 12 exercises and duration (minutes) of PT provided in total and for specific exercises and functional exercises. No changes were made to trial outcomes after the trial commenced.

### Data analysis

The sample size was determined based on the primary outcome (change in MVT knee-extension strength, fractured limb (F) % of non-fractured limb (NF), from baseline to follow-up) and calculated to detect a between-group difference of 20% (20% less strength deficit with ST) based on our two previous uncontrolled studies [1,20]. The magnitude of this difference is larger than what we consider the minimal clinically important difference at this time point, estimated to 10% based on our experience and given an average of five ST sessions. Accordingly, the study was considered exploratory, powered to detect changes in no other but the primary outcome and, given the positive results with ST, we considered it necessary to verify this in at least one (phase-3 like) confirmatory trial [36]. To be able to establish this effect according to Lehr’s formula and a mean (SD) group difference of 20% (30) in a two-sample *t*-test, 36 patients needed to be included in each group using a standard of 80% power and two-sided type 1 error rate 5%. To allow for a dropout rate of 20%, a total of 90 patients were included.

### Statistical analysis

All data analyses were blinded and based on a pre-defined statistical analysis plan. Data from primary, secondary and supplementary outcomes were validated by double entry. Less than 1% errors were located and corrected. The analysis of our primary outcome followed the intention-to-treat principle (ITT) based on data from all randomised participants. This analysis included participants who completed less than the full dose of the ST programme or outcome assessments according to protocol. For participants with missing primary outcome data, we performed multiple imputation assuming the data to be missing at random, validated by Little’s MCAR test. A total of five imputations were made in SPSS (version 22) based on the Monotone regression method. The linear regression one-way ANOVA model was used for each of the five datasets to analyse between-group differences in the change of fractured limb % of non-fractured limb (MVT F%NF) from baseline to follow up and the single pooled estimate was used for the primary statistical analysis. The same procedure was conducted as primary statistical analysis of the secondary outcome, the TUG test. Only two participants in this analysis had imputation performed and only of the TUG follow-up value (N = 75). Secondary analyses of all outcomes were conducted according to the per-protocol principle using complete data only. An analysis of covariance with adjustment for baseline scores (MVT F%NF) was performed to account for imbalances between groups at baseline. Between-group differences in change scores for unadjusted and adjusted baseline scores are both reported, as recommended by the CONSORT group [37].

All descriptive continuous data were examined for normality of distribution using Q-Q plots. Data are presented as means (1 SD) when normally distributed, otherwise as medians (first-third quartile), or as counts with percentages. Differences between groups were examined using the chi-square or Fischer exact test for categorical data and the Students t-test or the Mann-Whitney-U test for continuous data, as appropriate. Changes within groups from baseline to follow-up were determined by the paired samples T-Test or Wilcoxon Signed Rank Test as appropriate. All analyses were conducted with SPSS statistical software (SPSS statistical software version 22; SPSS inc. Chicago, Illinois, USA).

## Results

### Flow of participants

**Fig 1:**
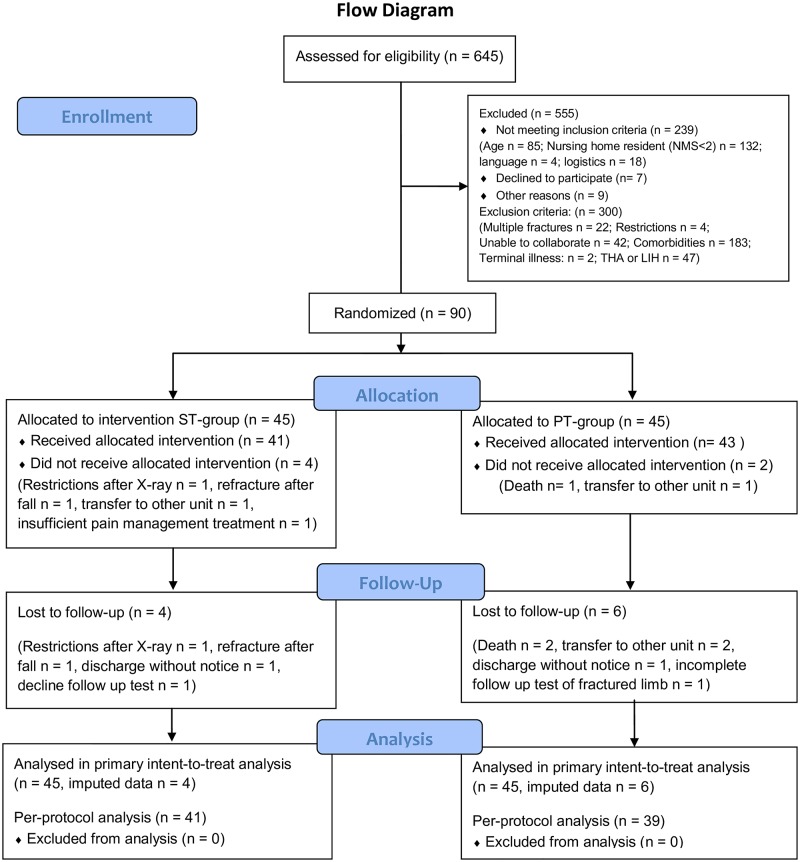
CONSORT flow diagram. ST = strength training group, PT = physiotherapy group.

Between October 2013 and May 2015, a total of 645 patients were examined for eligibility. A total of 90 out of the 106 eligible patients agreed to participate and were included in this study (Fig 1). No statistical difference was found in the distribution of fracture types between the groups (Table 1, S2 Table).

**Table 1:** Baseline characteristics of participants.

Characteristic	Randomised (n = 90)	
Group	ST (n = 45)	PT (n = 45)
**Participants**		
Age *(yr)*, mean (SD)	79.8 (7.7)	79.3 (7.5)
Men, n (%)	19 (20)	12 (27)
Women, n (%)	36 (80)	33 (73)
Body weight *(kg)*, mean (SD)	65.2 (13.5)	63.3 (13.9)
ASA Score, median (min-max), 1–4 score	2 (1 to 3)	2 (1 to 3)
NMS, median (IQR), 0–9 score	9 (7 to 9)	9 (7 to 9)
MMSE (n = 81), median (IQR), 0–30 score	29 (27 to 30)	29 (27 to 30)
**Fall history, n (%)**		
Fall in own home	24 (53)	16 (36)
Fall in the street	14 (31)	20 (44)
Fall with bicycle	4 (9)	5 (11)
Other	3 (7)	4 (9)
**Fracture type, n (%)**		
Femoral neck fracture	18 (40)	20 (44)
Trochanteric fracture	27 (60)	25 (56)
**Type of surgery, n (%)**		
Hemi-arthroplasty	17 (38)	19 (42)
Dynamic Hip screw, 2-hole	2 (4)	2 (4)
Dynamic Hip screw, 4-hole	2 (4)	3 (7)
Intra-medullary nail, short	22 (49)	18 (40)
Intra-medullary nail, long	2 (4)	3 (7)
**Discharge outcomes**		
Independent mobility at discharge, n (%)	39 (87)	39 (87)
Postoperative day of discharge, mean (SD)	11.6 (7.4)	11.8 (6.8)
**Discharge destination, n (%)**		
Own home	36 (80)	33 (73)
24h rehabilitation	7 (16)	6 (13)
Other unit	1 (2)	2 (4)
Nursing home	0	1 (2)
Died during admittance	1 (2)	3 (7)

ST = strength training group, PT = physiotherapy group, ASA score = American Society of Anaesthesiologists score, NMS = the modified New Mobility Score, MMSE = Mini Mental State Examination.

### Compliance with the trial protocol

**Fig 2:**
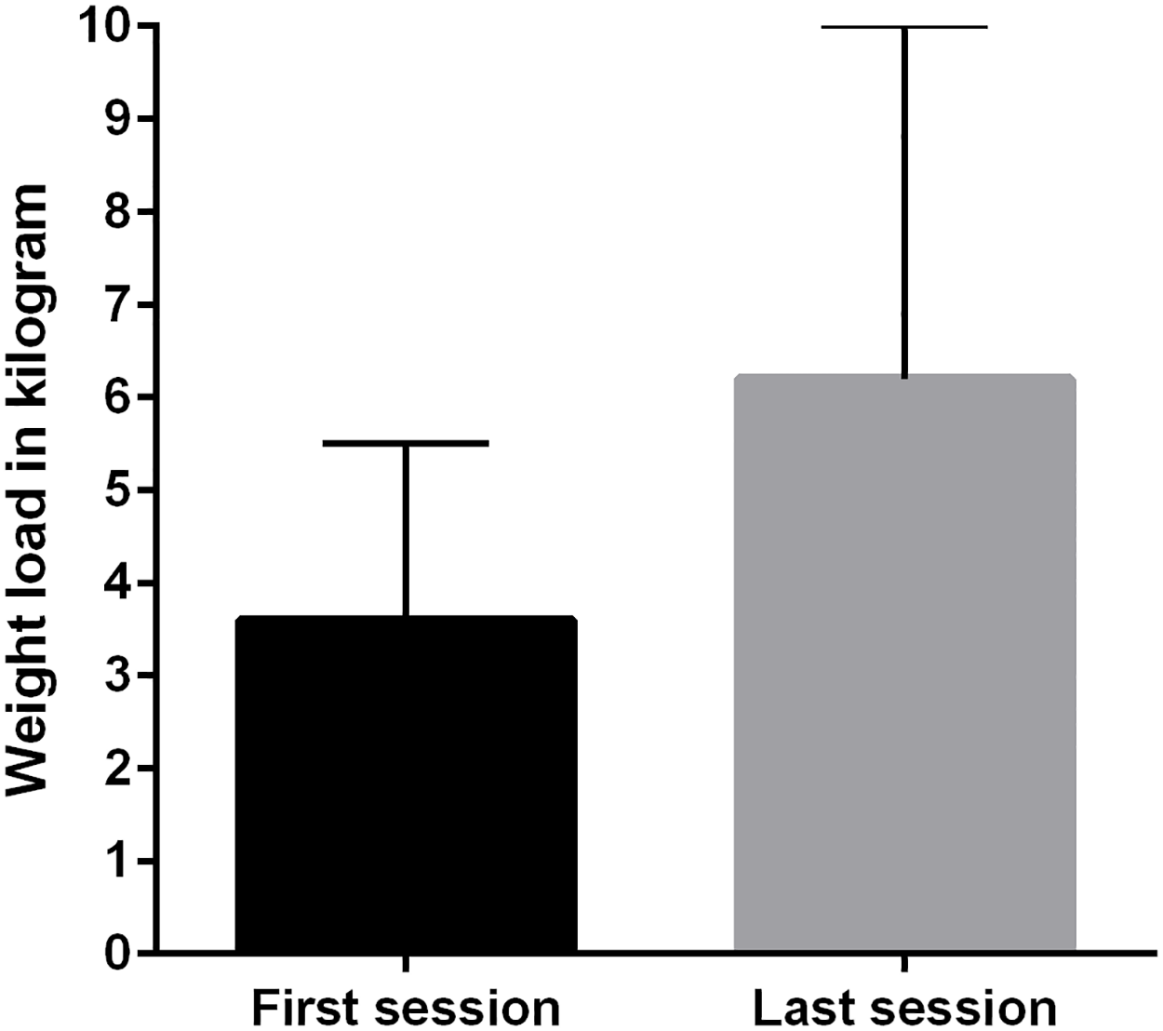
Mean (SD) weight load (kg) applied at first and last strength training session.

**Fig 3:**
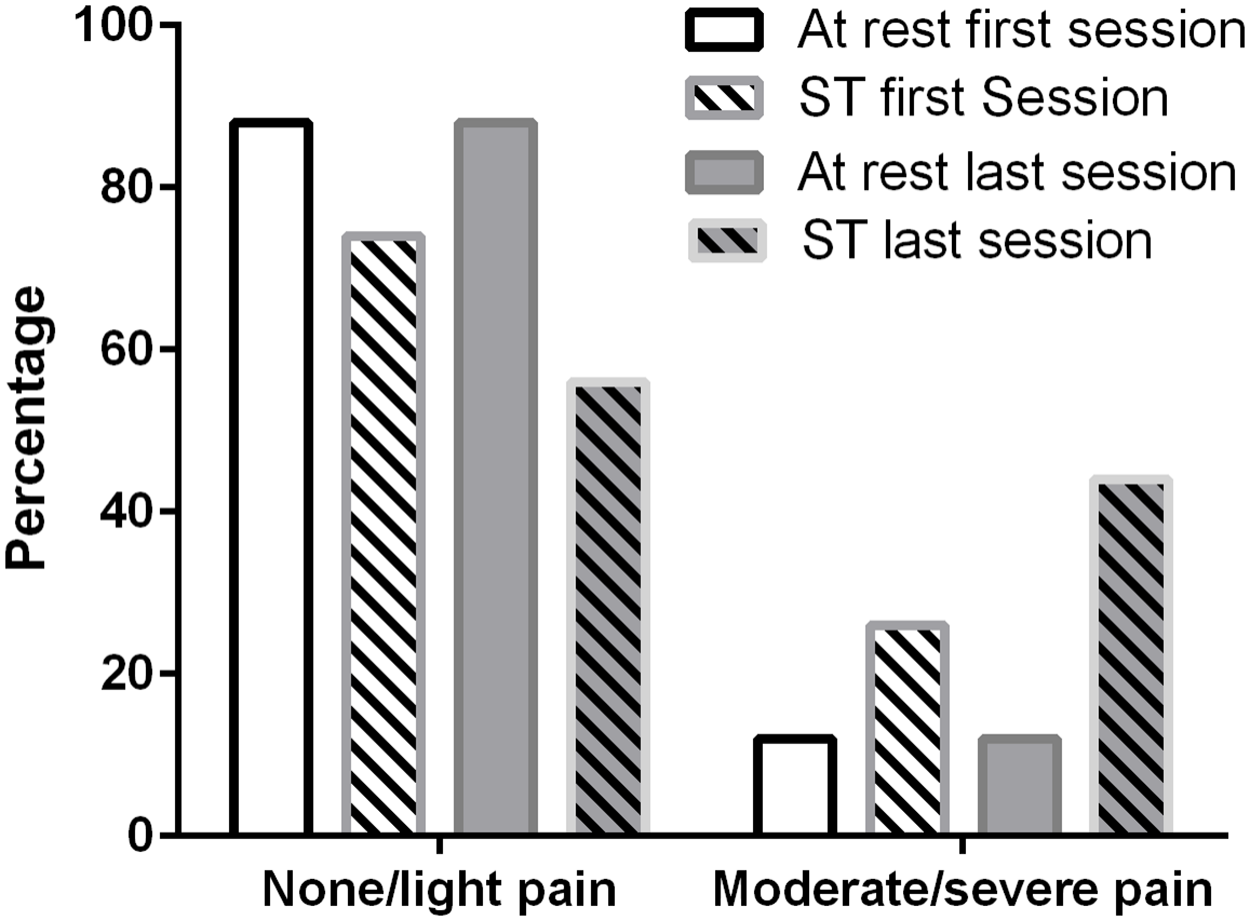
Percentage of patients with hip fracture-related pain recorded at rest and during strength training (ST) by Verbal Ranking Scale (None to intolerable pain).

The ST was conducted between postoperative day 2 and 8 (mean of 2.3 (0.8) days to 8.2 (2.9) days) and the participants completed a mean of 5.5 (2.8) ST sessions. The weight load increased significantly from a mean of 3.6 (1.9) kg at first session to 6.2 (3.8) kg in the last session (Table 2, Fig 2, S2 Table). A total of 80% of all planned ST sessions were completed. One missed session was seen in 16 participants and 2 (n = 7) or more (n = 5) in 12 participants. Limitations to completing a session or to perform better (more repetitions or increased weight load) were registered once per type of reported limitation. The primary limitations to completion of the ST were pain (47%), exhaustion (37%) nausea (5%), delirium (5%) or logistics (5%) (Table 2, S2 Table). Most participants (88%) reported no or light pain at rest before ST with no change in the proportion of patients from the first to the last ST session. At the first ST session, 26% (n = 11) reported moderate or severe pain during ST, while this was increased to 44% (n = 19) at the last ST session, which was a statistically non-significant change (Fig 3). Similarly, more than 80% (n = 73) reported no or light pain during the baseline test of knee-extension strength. Upon discharge, 77 (96%) out of 80 participants completing the program reported no or light pain at rest, while 69 (86%) patients reported no or light pain during the follow-up knee-extension strength test.

**Table 2:** Strength training outcomes.

**Outcome**	
Number of ST training days, mean (SD)	6.7 (2.9)
Completed ST sessions, mean (SD)	5.5 (2.8)
Missed ST sessions, mean (SD)	1.4 (1.8)
First postoperative day of ST, mean (SD)	2.3 (0.8)
Last postoperative day of ST, mean (SD)	8.2 (2.9)
Weight load first session *(kg)*, mean (SD)	3.6 (1.9)
Weight load last session *(kg)*, mean (SD)	6.2 (3.8)
Difference in weight load *(kg)*, mean (SD)	2.6 (3.4) ^a^
Change in percentage *(%)*, mean (SD)	104 (150)
**Limitations that affected the ST sessions (n = 38)**	
Pain, n (%)	18 (47)
Exhaustion, n (%)	14 (37)
Nausea, n (%)	2 (5)
Delirium, n (%)	2 (5)
Logistics, n (%)	2 (5)

ST = Strength Training.

^a^The Paired samples T-Test was used to determine the difference between weight loads in the first and the last ST session, *P* <.001.

The routine PT treatment was conducted over a mean of 5.8 (1.8) days. The mean training time per day was 22.1 (5.3) minutes, divided into functional training and specific exercises and included time spent on ST in the ST group (Table 3). A significant difference of 2.9 (95% CI 0.7 to 5.1) minutes of daily PT treatment was found between groups, explained by more time spent daily on specific exercises in the PT group than in the ST group (Table 3, S2 Table). The blinding of the outcome assessor was successful due to the blinding of both baseline results and patient allocation. No adverse events related to the protocol were reported. Twelve participants experienced a non-standardised treatment course during their admission such as isolation due to infection, transferral to cardiac or gastric units, death or discharge without notice to principal investigator. They did not differ from the cohort at baseline.

**Table 3:** Outcomes of physiotherapy treatment (PT).

Outcome	Groups		Difference between groups^a^
	ST (n = 43)	PT (n = 44)	ST minus PT
Number of physiotherapy training days, mean (SD)	6.0 (1.9)	5.6 (1.7)	0.34 (-0.4 to 1.1)
Total training time per day *(min)*, mean (SD)	20.6 (5.8)	23.5 (4.3)	-2.9 (-0.7 to -5.1)^b^
Functional training per day *(min)*, mean (SD)	13.6 (4.5)	14.3 (3.6)	-0.7 (-2.5 to 1)
Specific exercise training per day *(min)*, mean (SD)	7.0 (3.3)	9.3 (2.3)	-2.3 (-3.5 to -1.0)^c^

ST = Strength training group, PT = physiotherapy group.

^a^The Independent samples t-test was used to determine differences between groups.

^b^*P* = .011

^c^*P* <.001

### Effect of the interventions

#### Primary analysis

**Fig 4:**
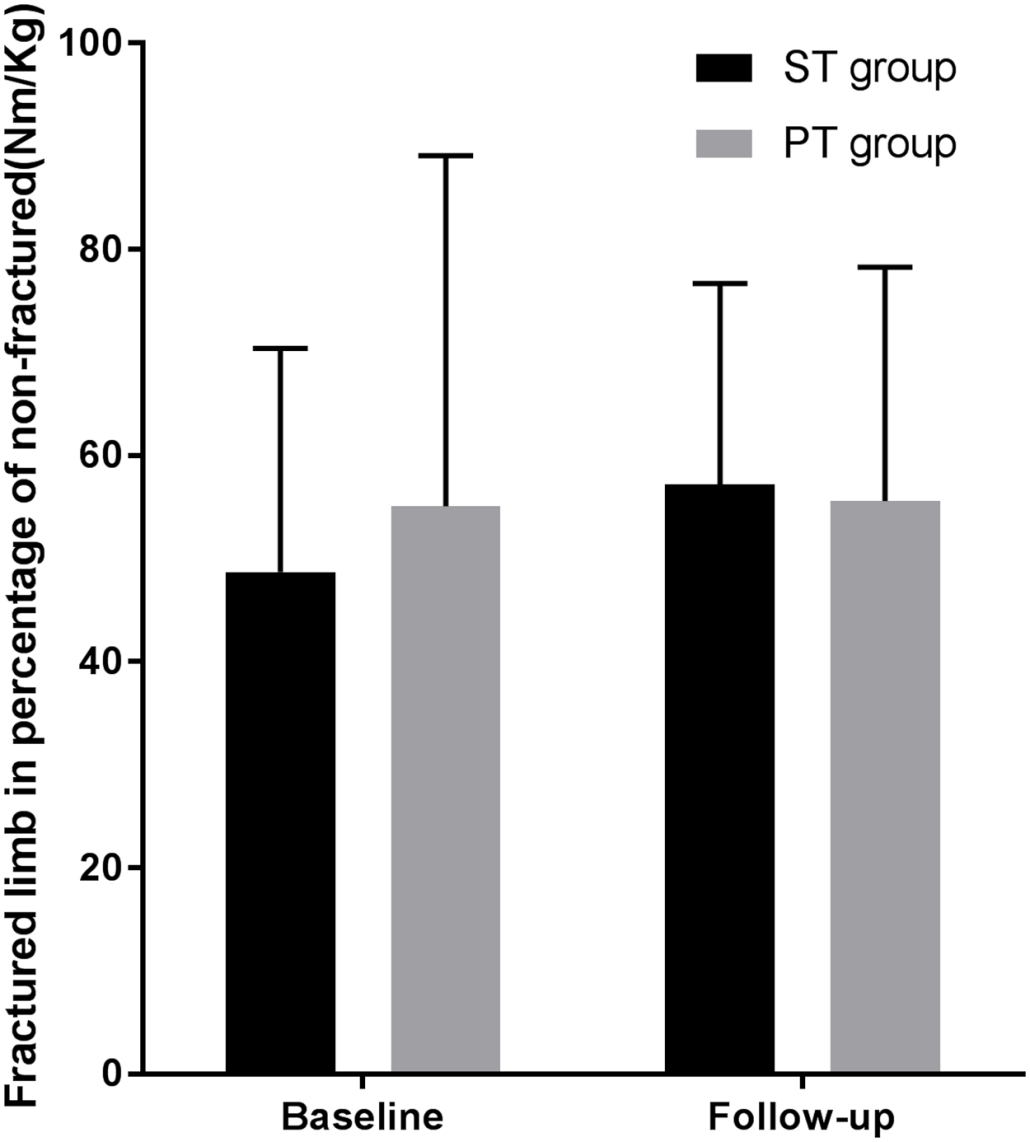
Mean (SD) fractured limb strength (MVT, Nm/kg) in percentage of the non-fractured limb for strength training group (ST group) and physiotherapy group (PT group).

**Fig 5:**
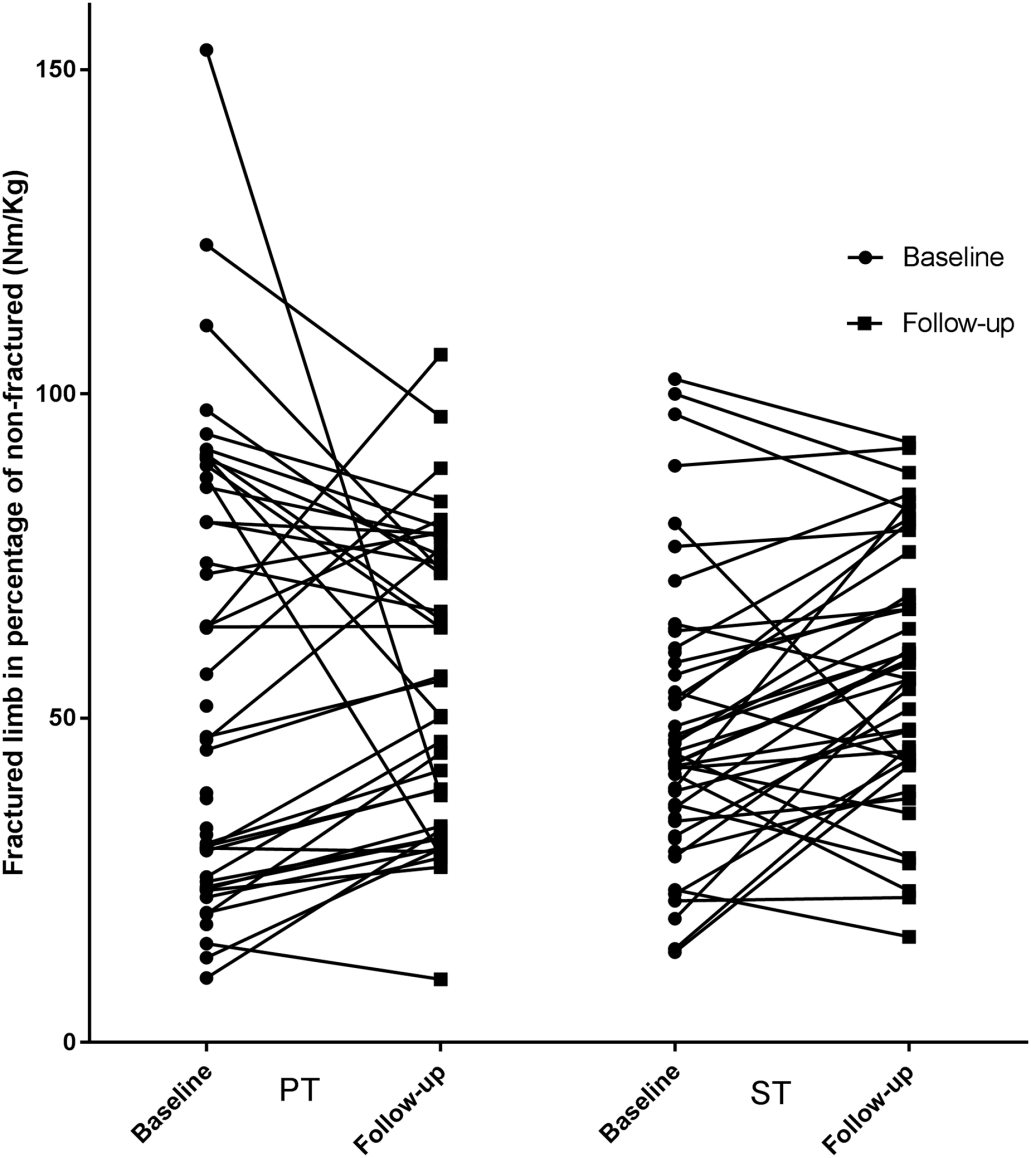
Change in the primary outcome (MVT Nm/kg), fractured limb in percentage of non-fractured from baseline to follow-up, with (ST) or without (PT) strength training.

In the intention-to-treat analysis (N = 90) of between-group differences, the primary outcome (MVT F%NF) improved 8.1% (95% CI -2.3; 18.4) by additional strength training from baseline to follow-up (Table 4, Fig 4, S2 Table). Adjusted for baseline differences (MVT F%NF) the result remained of no statistical significance (3.4% (95% CI -4.8 to 11.7)). In the per-protocol analysis of the primary outcome using complete data only (N = 80) a significant improvement of 10.5% (95% CI 0.3 to 20.7) was found in favour of additional ST. When adjusting for one outlier in the PT group, there was no significant difference between groups (7.5% (95% CI -1.0 to 16.1)). In addition, when adjusting for baseline differences there was no significant difference in the change of MVT F%NF between the groups (5.5% (95% CI -2.2 to 13.2)). The outlier detected in the PT group had an extreme negative change of 115% in the primary outcome (MVT F%NF) from baseline to follow-up. This was caused by an extreme positive baseline value of 153% due to motor block of the non-fractured limb at the baseline test (Fig 5). After performing the primary analysis, the analyses were repeated without the baseline value of this case, thus imputed in the ITT analysis, but excluded from the per-protocol analysis (n = 79) (Table 4, S2 Table).

**Table 4:** Table of primary and secondary results.

Outcome	Groups				Difference within groups		Difference between groups^a^
	Baseline		Follow-up		Follow-up minus Baseline		Follow-up minus Baseline
**Primary outcome**	ST (n = 45)	PT (n = 45)	ST (n = 45)	PT (n = 45)	ST (n = 45)	PT (n = 45)	ST minus PT
*Intention-to-treat*							
MVT F%NF *(%)*, mean (SD)	48.7 (21.7)	55.1 (34)	57.2 (19.5)	55.6 (22.7)	8.4 (16.7)^b^	0.02 (27.9)	8.1 (-2.3 to 18.4)
*Per protocol (n = 80)*	(n = 45)	(n = 45)	(n = 41)	(n = 39)	(n = 41)	(n = 39)	
MVT F%NF *(%)*, mean (SD)	48.7 (21.7)	52.9 (30.9)	56.9 (20)	55.7 (23.4)	8.0 (16.9)	-2.4 (28)	10.5 (0.3 to 20.7)
^c^1 outlier removed (n = 79)					8.0 (16.9)	0.53 (21.3)	7.5 (-1.0 to 16.1)
Postoperative day of test, mean (SD)	1.7 (0–7)	1.6 (0.7)	8.6 (2.0)	8.7 (1.6)	6.9 (2.0)	7.2 (1.7)	
MVT NF *(Nm/kg)*, mean (SD)	1.05 (0.44)	1.02 (0.48)	1.09 (0.47)	1.11 (0.38)	0.02 (0.22)	0.08 (0.39)	-0.06 (-0.08 to 0.2)
MVT F *(Nm/kg)*, mean (SD)	0.48 (0.23)	0.51 (0.34)	0.60 (0.30)	0.62 (0.35)	0.12 (0.22)	0.08 (0.20)	0.04 (-0.14 to 0.05)
**Secondary outcome**	(n = 38)	(n = 38)	(n = 37)	(n = 37)	(n = 37)	(n = 37)	
Postoperative day of test, mean (SD)	5.7 (2.4)	5.8 (1.9)	8.6 (2.0)	8.7 (1.6)	2.7 (1.8)	2.8 (1.9)	
TUG time, unadjusted *(s)*, mean (SD)	31.7 (12.5)	33 (14.5)	25.4 (11.8)	23.9 (9.6)	-6.4 (7.2)^d^	-9.3 (10.1)^d^	3.0 (-1.1 to 7.1)
TUG time, adjusted for baseline *(s)*, mean (SD)							2.4 (-0.8 to 5.6)

ST = Strength training group, PT = physiotherapy group, MVT = Maximal Voluntary Torque knee-extension, F = Fractured limb, NF = Non-fractured limb, MVT F%NF = Fractured limb MVT in percentage of non-fractured limb MVT, TUG = Timed Up and Go test.

^a^ Regression analysis of change in MVT F%NF and TUG from baseline to follow-up in percentage (CI) and seconds (CI) respectively and Independent samples t-test of changes in MVT F and NF.

^b and d^ Paired-samples T-test of change from baseline to follow-up within groups, ^b^MVT F%NF = *P* <.001 and ^d^TUG time = *P* <.001.

^c^1 outlier baseline MVT value for non-fractured limb in the control group excluded.

Subgroup analyses based on non-missing data and type of fracture showed a 10% (95% CI 2 to 17) between-group mean difference in reduction of strength deficit in favour of ST in the group of patients with trochanteric fractures compared to a 5% (95% CI -3 to 12) between-group mean difference within patients with a femoral neck fracture. However, the between-group analysis showed no statistical significant evidence of differential effects. To this, the female participants in this study were found to have mean knee-extension strength of 0.9 Nm/kg in the non-fractured limb and 0.5 Nm/kg in the fractured limb at follow up which has implications for risk of mobility limitation. Furthermore, subgroup analyses of participants with increased versus reduced strength deficit at follow-up showed non-significant tendencies of relations between older age, lower pre-fracture NMS and negative change in the strength deficit from baseline to follow-up. No single outcome could significantly explain the direction of change in strength deficit at follow-up with or without ST (Fig 5).

### Secondary analysis

The majority of participants (87%) achieved independent mobility before discharge and did this at a mean of 5.7 (2.8) days postoperatively. Only participants who achieved independent walking skills were assessed with the TUG test. For the participants assessed the TUG test result improved significantly from baseline to follow-up for both groups (n = 74, mean -7.8 (95% CI -5.8 to -9.9) seconds) but no statistical significant differences in improvement were found between groups (mean 3.0 (95% CI -1.1 to 7.1) seconds) (Table 4).

Walking speed measured by 10MWT at follow-up reached a mean of 0.54 (0.21) m/s for 76 participants. Fear of falling at follow-up measured by the ShortFES-I, was found to a mean score of 13.7 (5.5) point, equal to a moderate to high fear of falling. The 24-hour activity was measured between median postoperative day 1.5 (1–2) and 9 (7–10) with a median number of 6 (5–8) 24-hour days of activity data. The median 24-hour upright time was 62 (42–103) minutes per day and increased by a median of 61 (18–112) mins during the first postoperative week; from 22 (11–48) minutes to 92 (48–156). No significant differences between groups were found in any of these functional performance outcomes.

## Discussion

No significant between-group difference in the change of the strength deficit was found in the primary analysis, although the per-protocol analysis based on participants with complete data showed a significant and potentially clinically meaningful improvement of strength deficit by 10% during 5–6 sessions with PT and ST compared to PT only. However, the additional ST sessions did not improve functional performance or physical activity measured in upright time.

In this study it was not possible to reproduce the positive level of change in MVT F%NF achieved in the feasibility study (18%) [20], probably explained by several factors. First, the PT group received exercises similar to the routine PT exercises provided in the ST group, just without any form of strength training involved, as described in S2 Text, since this is the standard protocol for patients admitted to the unit. Thus, the PT group was no non-treatment group, but considered a control group to the intervention of additional strength training only. Secondly, the feasibility study showed that the patients tolerated weight loads well and thus the physiotherapist increasingly challenged the patients during this intervention and without any adverse events. This knowledge may have led to the considerably heavier weight loads applied in this study, which potentially caused the higher frequency of moderate or severe pain during ST. Nonetheless, hip related pain in the postoperative period is an already known limitation to acute phase rehabilitation and walking after HF surgery [21,38]. Finally, the ST was conducted as daily sessions, weekends included, as opposed to only on weekdays in the feasibility study. This means less opportunity for restitution between sessions and may have contributed to the less favourable performance outcomes of the intervention. A recent meta-analysis by Peterson et al. supports this by positive effects found in older adults with less weekly ST sessions [39]. This change of protocol from the previous study [20] was adapted in the expectation that more ST sessions would increase effectiveness of the intervention. On the contrary, this alteration may have reduced the effectiveness of the present intervention compared to the protocol of ST provided only on weekdays [20].

Cut-points for knee-extension strengths (MVT Nm/kg) and risk of severe mobility limitation have been examined by Manini et al. who found that values <1.01 Nm/kg for women and <1.13 Nm/kg for men were related to a high risk of subsequent mobility limitation [40]. According to cut-points by Manini et al., our participants in total, with mean knee-extension strength of 1.1 Nm/kg in the non-fractured extremity at follow-up, are at high risk of further mobility limitation [40]. This is especially expressed in the female participants in this study who were at high risk of mobility limitations based on the postoperative knee-extension strength deficit. Correspondingly, the level of fast gait speed of our participants, at a mean of 0.54 (0.21) m/s, was below the threshold 0.60–0.70 m/s associated with a strong risk of poor health outcomes [1,41,42]. These findings correspond well with previous findings linking strength deficit with poor physical performance in the acute phase after HF surgery [1]. Combined with the moderate to high fear of falling, our results indicate the need of further community-based rehabilitation including ST for patients with a HF [5,17]. This was tested in a study by Overgaard et al. [8] where positive results were found on improvement of gait speed to above threshold levels along with significant improvements of knee-extension strength, by addition of 6 weeks of progressive strength training in the community-based rehabilitation [8]. The standard error of measurement in TUG in patients with HF has been established to 3.5 seconds [30] which places the mean change of -7.8 (8.9) seconds from the first to the last TUG test as found in this study beyond the threshold. Therefore, although no between-group difference in the TUG-changes was observed, we consider both groups to have a clinically relevant improvement (Table 4).

The subgroup analysis on fracture type showed large strength deficits at follow-up, especially in the trochanteric group, but our results indicate an increased benefit of ST in the subgroup of patients with trochanteric fractures (10%) compared to femoral neck fractures (5%). This calls for an increased focus on ST for patients with trochanteric fractures, as strength deficits are associated with larger functional deficits after HF surgery in general [1,8].

A limitation to translation of our findings is the limited sample size and the selected population, which excludes, for example patients with low cognitive status and physical function. However, we do not see any limitations transferring the ST treatment as a modality into the rehabilitation of these patients, as supported by recent reviews [17,43]. The dosage of ST may have been too low to induce sufficient impact on the knee-extensor muscles to show a change within 5–6 sessions [39]. However, as the weight loads applied and the frequency of pain related to ST was increased, we believe that the dose applied was up to a maximum for our participants without compromising patient motivation to participate, as supported by Milte et al. [44]. The intervention period was short and only very limited changes in strength may be expected during such short time span. Pain during PT was not reported systematic, which is a limitation to the comparison of treatment-related pain between groups. Finally, a small but significant between-group difference in the PT time provided in favour of the PT only group may have influenced the results. The difference was seen primarily on the time spent on specific exercises, which could reflect the therapists’ choice but also the participants’ lack of ability to participate in lengthy exercise sessions in the acute setting. Thus, a therapeutic estimate to target the exercise within the limits of the participants’ resources may explain this significant, but to the pragmatic clinical setting, very little difference.

This study is to our knowledge the first to examine the efficacy of systematic ST conducted in the acute ward after HF surgery. Strengths of this study were high compliance with the randomised protocol, assessor blinding, an intervention that participants and the therapist were happy to participate in and the realistic setting of the intervention. The time consumption to conduct a ST session was estimated to be 15 minutes per day and performed as a part of the PT approach using only low cost equipment. This means that few resources are needed to conduct ST as performed in this study and with the potential to minimise the quadriceps’ strength deficit after surgery by 10% for those completing the planned dose as a part of the early PT. In the fragile population of patients with a HF in the acute phase, where the ability to participate in functional exercise is compromised, we consider this possibility to improve outcomes of clinical importance.

## Conclusion

PT with addition of 5 sessions of ST yielded no additional improvements compared to PT without ST in reducing the strength deficit at follow-up in patients with a HF. It is debatable whether larger improvements than the observed 8–10%, can be expected given that only five exercise sessions, on average, were completed. In fragile patients with a hip fracture in the acute phase, where the ability to participate in functional exercise is compromised, we still consider early strength training a possibility to improve outcomes of clinical importance, given the results of the per-protocol analysis. The present data provides an important basis and call for future investigations including longer term interventions.

## Supporting information

S1 Table CONSORT 2010 checklist of information to include when reporting a randomised trial. (DOC)

S2 Table Dataset of primary and secondary outcomes. (XLSX)

S1 Text Study protocol. (DOC)

S2 Text Appendix 1. PT treatment exercise guide for patients with a hip fracture.
(PDF)
