# Supplementary material for: Effectiveness of acute in-hospital physiotherapy with knee-extension strength training in reducing strength deficits in patients with a hip fracture: A randomised controlled trial
Source: PLoS One. 2017 Jun 29;12(6):e0179867. doi: 10.1371/journal.pone.0179867 (PMC5491058; doi:10.1371/journal.pone.0179867)
Supplement: S2 Text — PT treatment exercise guide for patients with a hip fracture. (PDF) [file pone.0179867.s004.pdf]

Hvidovre Hospital
**Department of Physio-
& Occupational Therapy**

Professionally responsible:
The Physiotherapists at Hvidovre Hospital

Exercise guide for patients with a hip fracture

The physiotherapist will outline the aim and plan for your rehabilitation. The exercises in this guide will be a part of the rehabilitation programme.

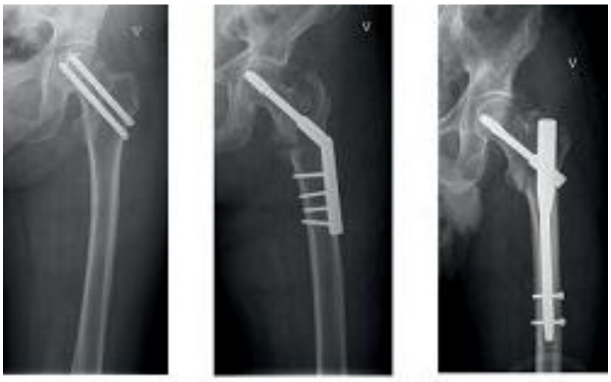

1. Parallel pins 2. DHS 3. IMHS

Which type of surgery have you had?

You have had surgery performed on your hip from one of the three types of surgery that you can see on the x-rays here on the left.

1, **Parallel pins:** Stabilises the fracture with two parallel pins.
2, **DHS:** Stabilises the fracture with a dynamic screw and brace.
3, **IMHS:** Stabilises the fracture with an intra-medullary nail.

The physiotherapist will discuss which type of surgery you have had and what this means for your rehabilitation.

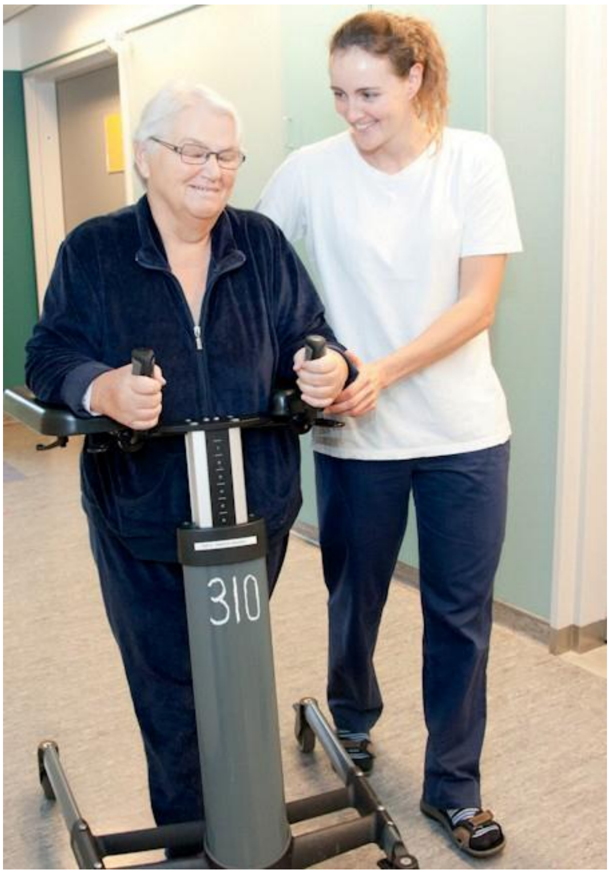

Getting started on the rehabilitation

It is important that you start using your operated leg as soon as possible. The physiotherapist will initially help you with the exercises until you are able to exercise on your own.

It is very important to exercise even though pain cannot be fully avoided when initially using your operated leg.

You will be able to perform more and more of the exercises as you regain your strength, mobility and balance. Your physiotherapist will progressively guide you through the relevant exercises.

Hvidovre Hospital
Department of Physio- & Occupational Therapy, 234 / 236
Direct telephone: +45 3862 2232

Hvidovre Hospital
Kettegård Allé 30
2650 Hvidovre
www.hvidovrehospital.dk
Telephone: +45 3862 3862

REGION H
November 2014

Exercises

It is very important that you also stay physically active when you are lying in your bed. This will help you to a faster recovery and in getting up and about again. You can do the first three exercises in this guide during the day or night. These exercises help to stimulate your blood circulation and reduce the loss of muscle strength in your legs.

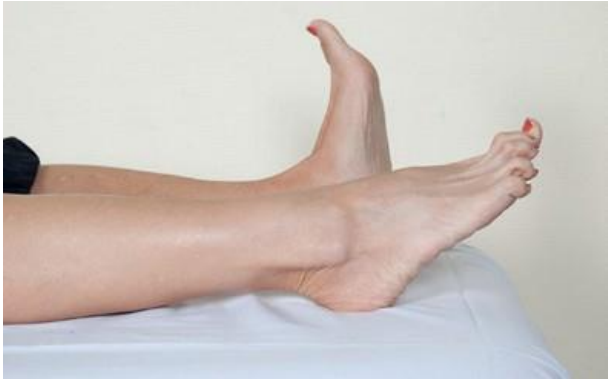

Exercise 1

The exercise stimulates the blood circulation in your legs.

Lie on your back with straight legs.

- Flex your feet up and down at the ankles.

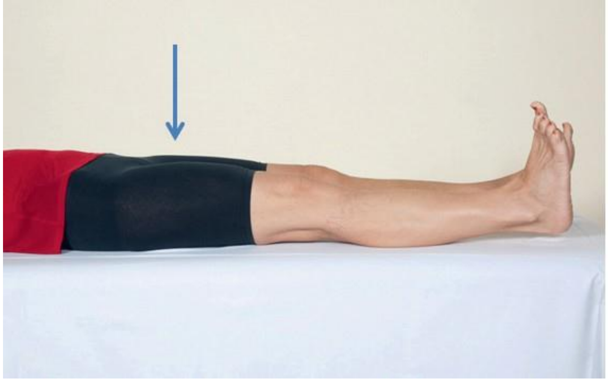

Exercise 2

The exercise reduces the loss of muscle strength in your thighs.

Lie on your back with straight legs.

- Tighten the muscles at the front of your thighs so that your knees press down on the mattress.
- Hold the tension, count slowly to 10 and relax.

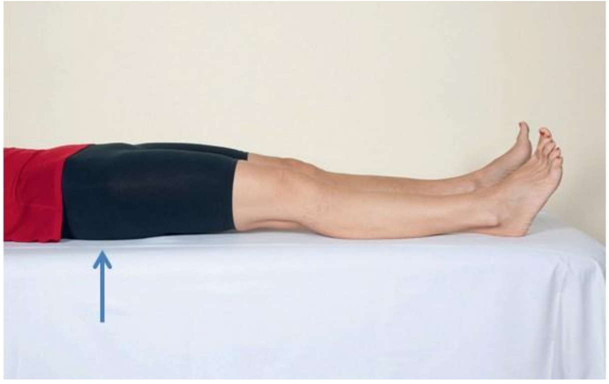

Exercise 3

The exercise reduces the loss of muscle strength in your buttocks.

Lie on your back with straight legs.

- Clench your buttocks.
- Hold the tension, count slowly to 10 and relax.

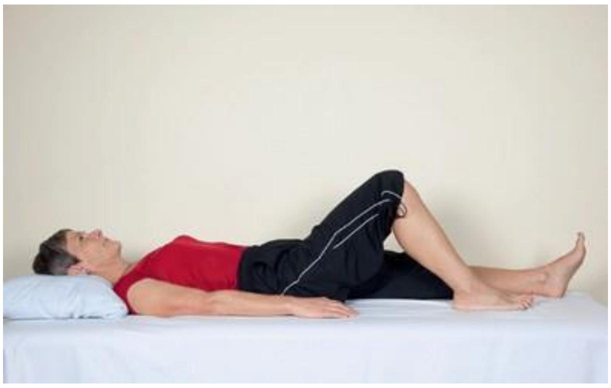

Exercise 4

The exercise increases the mobility of your hip and knee.

Lie on your back with straight legs. Pull in your navel.

- Bend your operated leg at a steady pace by letting the heel slide along the surface.
- Stretch out your leg again and release your navel.

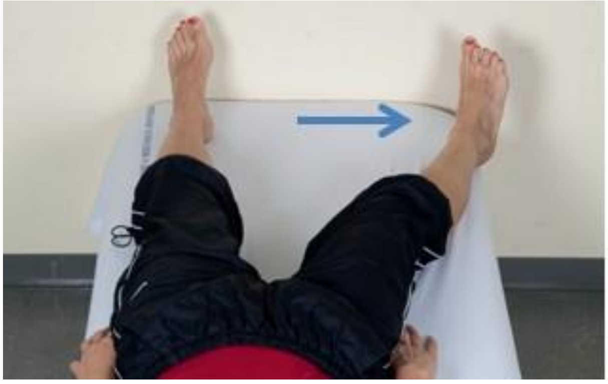

Exercise 5

The exercise increases the mobility in your hip.

Lie on your back with a little distance between your feet. Pull in your navel.

- Glide your operated leg out to the side by letting the leg slide along the surface. Toes should be pointed towards the ceiling.
- Glide your leg back to the middle.

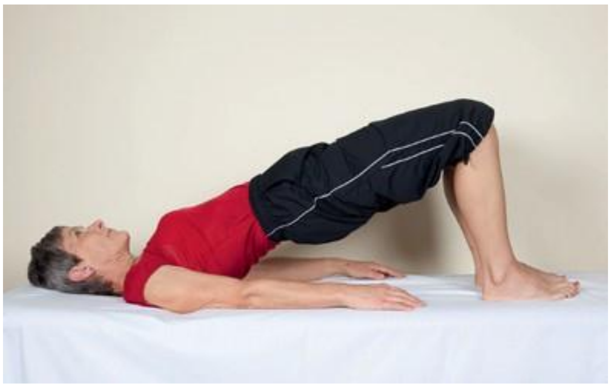

Exercise 6

The exercise strengthens the muscles in your buttocks and backside of the thighs.

Lie with knees bent and feet in the surface. Pull in your navel.

- Clench your buttocks and lift your pelvis from the surface.
- Hold this position for a moment, lower back down slowly and relax.

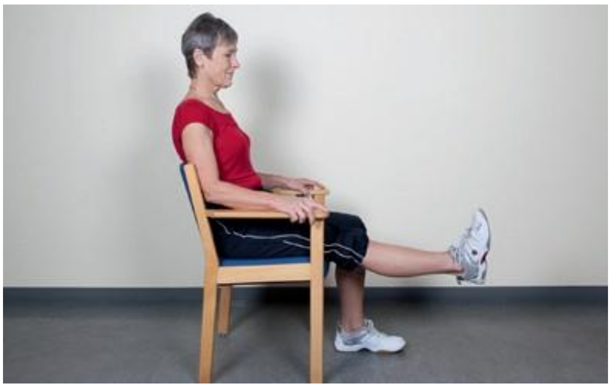

Exercise 7

The exercise increases the strength in your thighs and mobility in the knees.

Sit back in the chair. Straighten your knee fully in your operated leg.

- Hold this position, count slowly to 10 and lower your leg back down slowly.

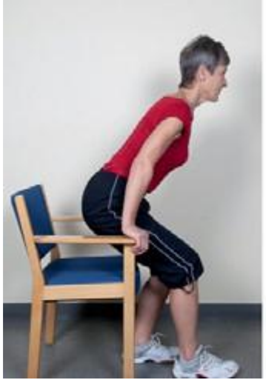
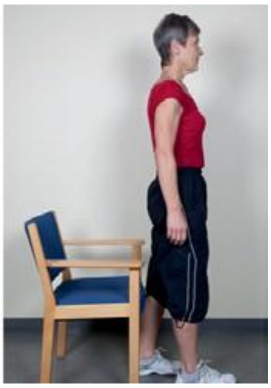

Exercise 8

The exercise increases your balance and the strength in your legs.

Sit on a chair with armrests. Move to the front of the chair and lean your upper body slightly forward towards your knees.

- Stand up, perhaps with support from the armrests.
- Sit back down supported by the armrests.

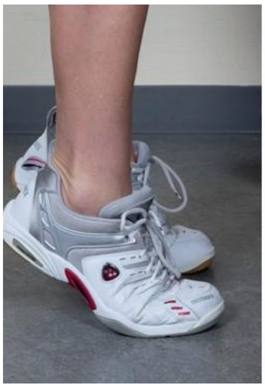
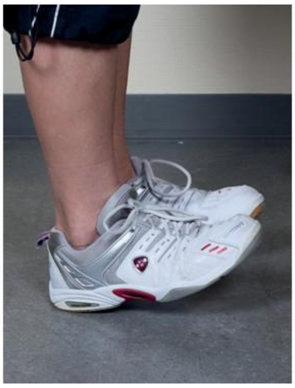

Exercise 9

The exercise improves the blood circulation in your legs, increases the strength in your lower legs and mobility in your ankles.

Support yourself on a footboard or a table if needed. Stand with equal weight on both legs. Feet must be pointing straight forward.

- Rise on your toes and lower down slowly.
- Tilt back on your heels and forward again.
- Remember to keep your back straight.

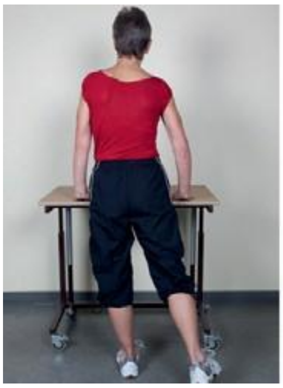
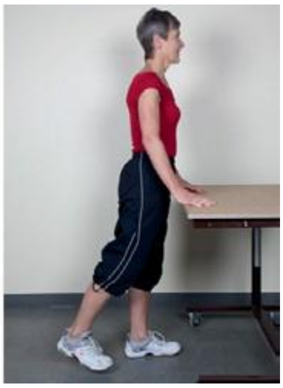

Exercise 10

The exercise increases your balance and the muscle strength around your hip.

Support yourself with a footboard or a table if needed. Stand with your weight on the healthy leg. Pull in your navel.

- Push the operated leg diagonally backwards.
- Hold this position for a moment and pull the leg slowly forward again.
- Repeat the exercise with the opposite leg.

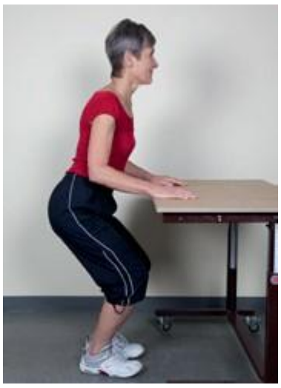

Exercise 11

The exercise increases the strength in your thighs.

Support yourself on a footboard or a table if needed. Stand with equal weight on both legs. Your feet must be pointing straight forward. Pull in your navel.

- Bend down in your knees, knees directly over your feet and rise slowly back up.

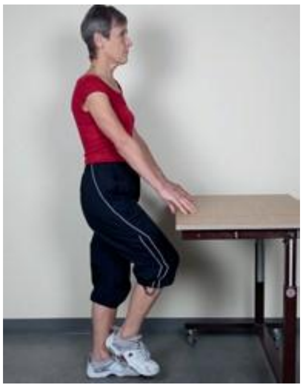

Exercise 12

The exercise increases your muscle strength in the front of your thighs and around the hip.

Support yourself to a footboard or a table if needed. Stand with your weight on the healthy leg. Pull in your navel.

- Lift your operated leg up towards your stomach with a bent knee, lower the leg back down slowly again.
- Repeat the exercise with the opposite leg.

Good advice

Exercise

The exercises may make your muscles sore and tired, but if you experience pain during the exercises, it should decrease within a few hours.

You are supposed to continue to do the exercises in this exercise guide after discharge.

Car and bicycle

You are allowed to drive a car and a bicycle according to the Road Traffic Acts. However, you must be able to fully control the pedals and be able to perform an emergency stop. We recommend that you ask your insurance company if they have any special regulations in this regard.

Cardio exercise

Daily cardio exercise such as walking is good for you. Take several short walks in the beginning and slowly increase the distance to longer walks.

Stairs

Feel free to climb stairs as usual immediately after your surgery. In the case of pain or discomfort, we recommend the following:

- Up the stairs: Put the healthy leg up first.
- Down the stairs: Put the operated leg down first.
- If you are using a cane or a crutch, this must follow the operated leg.

Translated from original language by Lise Kronborg, Department of Physio- & Occupational Therapy, Hvidovre Hospital, Denmark.
https://intern.publikationer.regionh.dk/publication/f323a6c3-dd0e-4c44-a338-a1eb0097f0ea/traeningsguide-til-patienter-med-hoftebrud.pdf
